# Mechanical Conflicts in Twisting Growth Revealed by Cell-Cell Adhesion Defects

**DOI:** 10.3389/fpls.2019.00173

**Published:** 2019-02-25

**Authors:** Stéphane Verger, Mengying Liu, Olivier Hamant

**Affiliations:** Laboratoire de Reproduction et Développement des Plantes, ENS de Lyon, UCBL, INRA, CNRS, Université de Lyon, Lyon, France

**Keywords:** adhesion, twisting, mechanical stress, morphogenesis, arabidopsis

## Abstract

Many plants grow organs and tissues with twisted shapes. Arabidopsis mutants with impaired microtubule dynamics exhibit such a phenotype constitutively. Although the activity of the corresponding microtubule regulators is better understood at the molecular level, how large-scale twisting can emerge in the mutants remains largely unknown. Classically, oblique cortical microtubules would constrain the deposition of cellulose microfibrils in cells, and such conflicts at the cell level would be relaxed at the tissue scale by supracellular torsion. This model implicitly assumes that cell-cell adhesion is a key step to transpose local mechanical conflicts into a macroscopic twisting phenotype. Here we tested this prediction using the *quasimodo1* mutant, which displays cell-cell adhesion defects. Using the *spriral2/tortifolia1* mutant with hypocotyl helical growth, we found that *qua1*-induced cell-cell adhesion defects restore straight growth in *qua1-1 spr2-2*. Detached cells in *qua1-1 spr2-2* displayed helical growth, confirming that straight growth results from the lack of mechanical coupling between cells rather than a restoration of SPR2 activity in the *qua1* mutant. Because adhesion defects in *qua1* depend on tension in the outer wall, we also showed that hypocotyl twisting in *qua1-1 spr2-2* could be restored when decreasing the matrix potential of the growth medium, i.e., by reducing the magnitude of the pulling force between adjacent cells, in the double mutant. Interestingly, the induction of straight growth in *qua1-1 spr2-2* could be achieved beyond hypocotyls, as leaves also displayed a flat phenotype in the double mutant. Altogether, these results provide formal experimental support for a scenario in which twisted growth in *spr2* mutant would result from the relaxation of local mechanical conflicts between adjacent cells *via* global organ torsion.

## Introduction

Because complex morphogenesis generally involves differential growth, mechanical conflicts are widespread in developing organisms. In animals, such conflicts can be resolved through cell rearrangements, as cells are in principle free to move. Yet, cell-cell adhesion often prevents such outcome and patterns of tension and compression appear. Mechanical conflicts can be resolved through global tissue deformation, as shown for instance in the gut (Savin et al., 2011; Nerurkar et al., 2019). Interestingly, some of the relevant mechanotransduction factors play a role in cell-cell adhesion. For instance, cadherins are both central regulators of epithelial cohesions and transducers of mechanical signals inside the cell (Leckband and de Rooij, 2014). Therefore, while mechanical conflicts emerge from differential growth and cell-cell adhesion, they also in turn contribute to growth patterns and adhesion through the cell response to mechanical stress, in a feedback loop.

With few exceptions such as pollen tube and fiber cell growth (Gorshkova et al., 2012; Chebli and Geitmann, 2017; Marsollier and Ingram, 2018), cell-cell adhesion and thus symplastic growth is ubiquitous in developing plant organs. The presence of contiguous cell walls with a pectin-rich middle lamella maintains adhesion between adjacent cells (Jarvis et al., 2003) (Daher and Braybrook, 2015) Besides, many reports point at the high degree of growth heterogeneity in plant tissues (Hong et al., 2018). It follows that mechanical conflicts are widespread in growing plants. As reported in animals, plant cells are able to sense and respond to such cues to control cell division plane orientation (Lintilhac and Vesecky, 1984; Louveaux et al., 2016), growth direction (Green and King, 1966; Hamant et al., 2008), cell polarity (Heisler et al., 2010; Nakayama et al., 2012; Bringmann and Bergmann, 2017) and cell identity (Coutand et al., 2009; Landrein et al., 2015). In parallel to these active responses to stress, mechanical conflicts may also be resolved through passive and global tissue deformation (Coen et al., 2004). For instance, mechanical conflicts are thought to play a major role in shaping complex floral shapes, such as Antirhinum petals, as a result of instructive biochemical signals, but without necessarily involving an active mechanical feedback on cells (Coen and Rebocho, 2016; Rebocho et al., 2017). One of the challenges for future research in this area is to understand the relative contributions of passive and active responses to mechanical stress in morphogenesis. Here we take the example of organ twisting to explore that question.

Several mutations on microtubule regulators, or even on tubulins, lead to twisted organs in Arabidopsis (Ishida et al., 2007b; Smyth, 2016). In such mutants, cells exhibit oblique cortical microtubule orientations and the handedness of the microtubule helix is always opposite to the handedness of tissue growth (Ishida et al., 2007a). For instance, the *lefty* mutations in α-tubulins lead to both a left-handed helical growth and a right-handed cortical microtubule orientations in the root epidermis (Thitamadee et al., 2002). Such phenotypes are only partially understood.

What is best known is the relation between microtubule orientation and growth: except for a few counterexamples [e.g., (Himmelspach et al., 2003; Sugimoto et al., 2003)], cortical microtubules generally guide the deposition of cellulose microfibrils; as cellulose microfibril stiffness constrain cell growth direction, cortical microtubule orientation becomes a proxy for the mechanical anisotropy of cell walls. Therefore, right-handed microtubule orientations would inevitably drive cell growth direction in a left-handed helix (Thitamadee et al., 2002; Smyth, 2016). Conversely, organ twisting is affected when the cellulose synthase—microtubule nexus is impaired in the *csi1* mutant (Landrein et al., 2013). Another cell wall mutant has recently been shown to have organ twisting without affecting microtubule organization, but is nevertheless believed to impact cellulose organization and cell wall mechanical anisotropy (Saffer et al., 2017).

**Figure 1:**
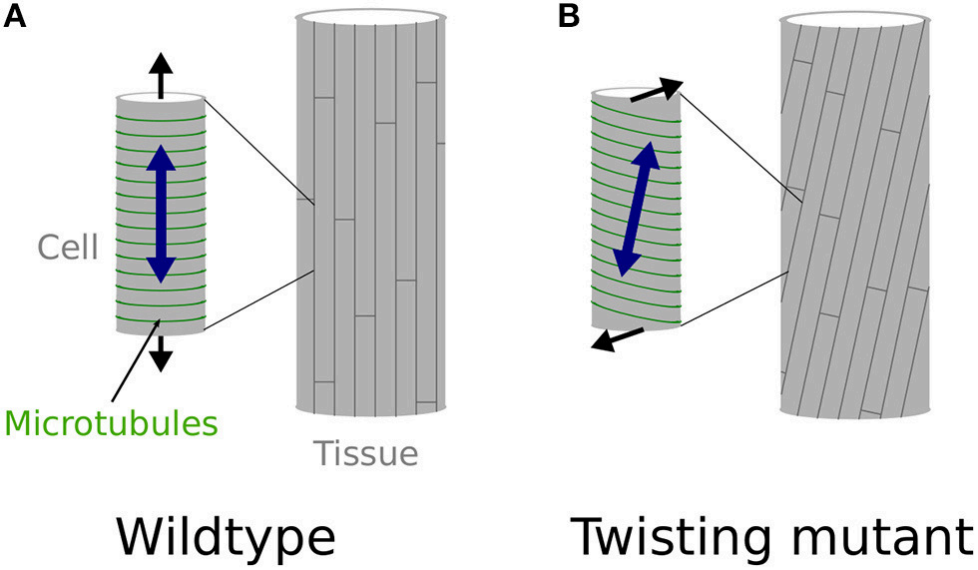
Twisting, from the molecular to the organ scale. **(A,B)** Schematic representation of the effect of cortical microtubules (represented in green) orientation at the cell level (small cylinder on the left) and its effect at the whole organ level (cylinder on the right), explaining straight, and twisting growth in a cylindrical organ. **(A)** Transverse cortical microtubules promote the longitudinal expansion of the cell, which leads to straight cell files at the organ level, as observed in wild-type hypocotyls, at least when considering inner cells (Crowell et al., 2011). **(B)** Tilted cortical microtubules impose a tilted mechanical anisotropy of the cell wall leading to the twisting of the cell at the single cell level. However, because cells are attached to one another they cannot twist by themselves and the mechanical conflict is relaxed through global organ torsion, as in *spr2-2* seedlings.

What is least known is 2-fold. First, it is unclear how microtubule arrays would acquire a stable and oblique orientation. Reports so far rather suggest that unstable microtubules tend to acquire a right-handed orientation (as in the *lefty* mutants), while stabilized microtubules acquire a left-handed orientation (Ishida et al., 2007b). This latter case is typical of the *spiral2/tortifolia1* mutant, which exhibits right-handed helical growth (Buschmann et al., 2004; Shoji et al., 2004). SPR2 was recently shown to bind and stabilize the minus end of microtubules to control their depolymerization rate, with an indirect impact on microtubule severing (Fan et al., 2018; Nakamura et al., 2018), although this latter point is debated and might depend on tissue identity (Wightman et al., 2013). In the end, microtubule dynamics are stimulated in *spr2* mutants, resulting in more stable cortical microtubule alignments. It remains unclear how affecting microtubules dynamics would lead to stable and consistent left or right handedness of cortical microtubule arrays. It has been proposed that the origin of such handedness lies in the microtubule structure itself. Microtubules are in general composed of 13 protofilaments and this confers them a straight structure. However, microtubules can in principle be composed of 10 to 16 protofilaments, some of these configurations conferring them a consistent left or right handed twisted structure (Pampaloni and Florin, 2008). Such chirality at the molecular level could be the basis for the consistent tilted microtubule arrays, however this has not been confirmed in twisting mutants so far (Ishida et al., 2007b). Second, it is unclear how local cell wall modifications would lead to torsion of a whole organ. Indeed, because they exhibit oblique mechanical anisotropy in their walls, each cell would simply twist around their axis as they grow, if they were not attached to one another (Wada and Matsumoto, 2018). However, because of cell-cell adhesion, these cells cannot twist independently. It has been proposed that such local mechanical conflicts could be relaxed by the global torsion of the organ (Wada and Matsumoto, 2018, see Figures 1A,B). However, the presence of these conflicts, and their role in helical growth, has never been demonstrated *in vivo*. This is what we aim at testing here.

## Materials and Methods

### Plant Material and Genotyping

The *qua1-1* (WS-4) T-DNA insertion line and the *spr2-2* (Col-0) EMS mutant, were previously reported in Bouton et al. (2002) and Shoji et al. (2004), respectively. The *qua1-1* mutant was genotyped using the primers described in Bouton et al. (2002) and the *spr2-2* mutant was genotyped by Sanger sequencing using the following primers: FW_5′-TGTCATCAGCAGCTCAGACA-3′ and RV_5′-TGAGAGAGTGGAACCATCGG-3′.

### Growth Conditions

*Arabidopsis thaliana* seeds were sown on solid custom-made Duchefa “Arabidopsis” medium (DU0742.0025, Duchefa Biochemie), containing either 1 or 2.5% agarose as gelling agent (**Figures 4K,L**, and see Verger et al., 2018).

Seeds were cold treated for 48 h to synchronize germination and then grown in a phytotron at 20°C. For hypocotyl etiolation, seeds were exposed to light for 4 h to induce germination. The plates were then wrapped in three layers of aluminum foil to ensure skotomorphogenesis, and placed in a phytotron at 20°C for 4 days before imaging.

### Cell Wall Staining and Confocal Microscopy

For cell wall staining, plants were immersed in 0.2 mg/ml propidium iodide (PI, Sigma-Aldrich) for 10 min and washed with water prior to imaging. For imaging, samples were placed between glass slide and coverslip separated by 400 μm spacers to prevent tissue crushing. Images were acquired using a Leica TCS SP8 confocal microscope. PI excitation was performed using a 552 nm solid-state laser and fluorescence was detected at 600–650 nm. Stacks of 1024 × 1024 pixels (pixel size of 0.363 × 0.363 micron) optical section were generated with a Z interval of 1 μm.

### Twisting Angle Quantifications and Statistical Analyses

**Figure 2:**
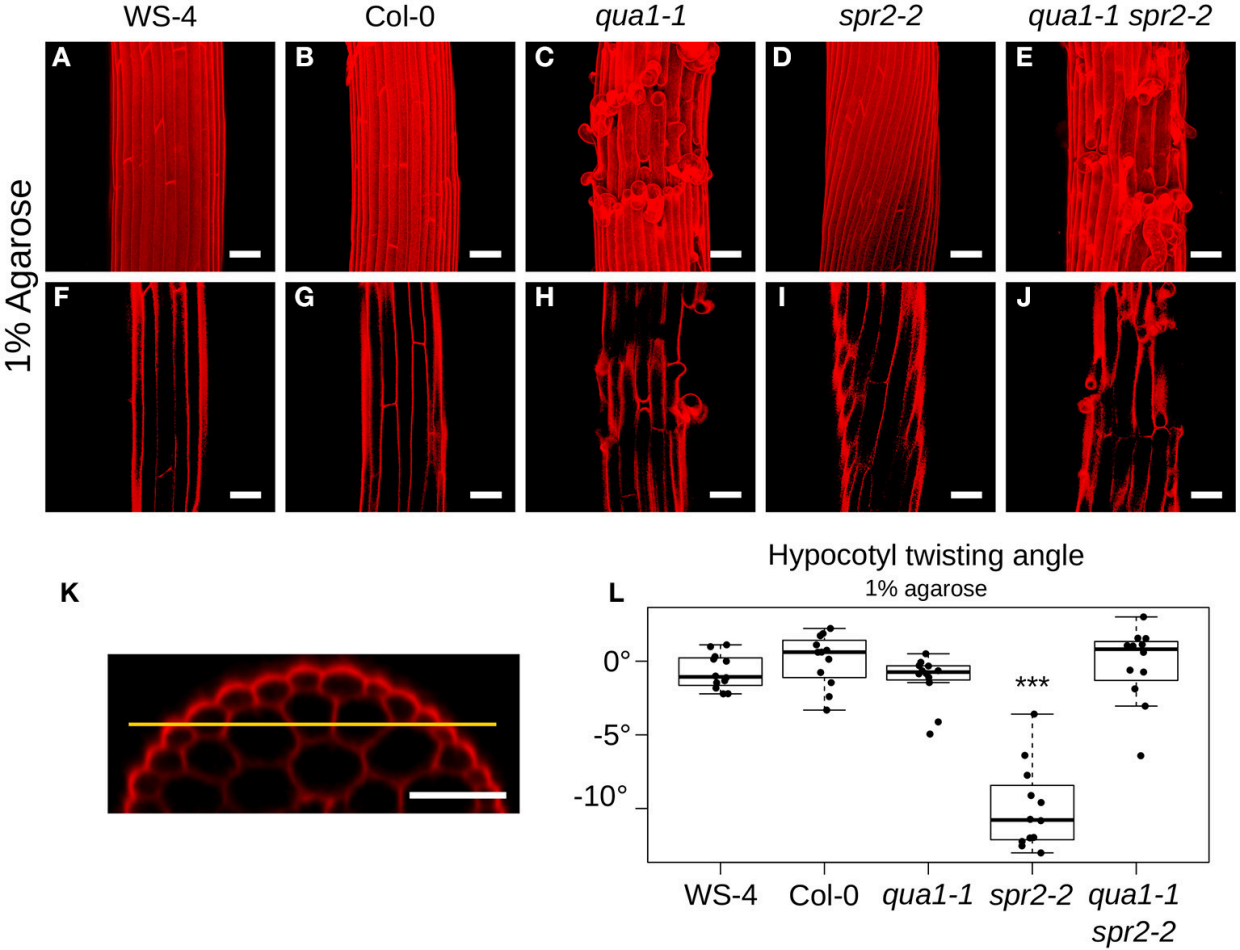
Loss of cell-cell adhesion prevents hypocotyl twisting in *qua1-1 spr2-2*. **(A–E)** Z-projections (maximal intensity) of confocal stacks from representative (12 samples observed in 3 biological replicates for each genotype/condition), propidium iodide stained, four-day old dark-grown hypocotyls. **(F–J)** Optical sections from the corresponding stacks from **A–E**, revealing the first cortex cell layer in the hypocotyl (i.e., the layer under the epidermis), following the yellow line drawn in **(K)**. **(K)** is an orthogonal section of an hypocotyl showing the epidermal as well as the two cortex cell layers. **(A,F)** WS-4, **(B,G)** Col-0, **(C,H)** *qua1-1*, **(D,I)** *spr2-2*, and **(E,J)** *qua1-1 spr2-2*, highlight the twisting phenotype of *spr2-2* as compared to the straight growth of the other genotypes. **(L)** Boxplot of twisting angle values, representing each data point and their distribution for each genotype. An angle of 0° corresponds to no twisting (straight growth), while positive and negative angle values mark left-handed and right-handed twisting, respectively. Wilcoxon rank sum test ^***^p < 0.0005. Scale bars, 50 μm.

We quantified the angle of cell files of the first cortex cell layer in the hypocotyl (i.e., the layer under the epidermis, Figure 2K). For each condition/mutant we quantified the twisting angle of 12 hypocotyls from 3 biological replicates. The angles were measured relative to the hypocotyl axis. An angle of 0° reveals no twisting, while positive and negative angle values mark left-handed and right-handed twisting, respectively. Twisting angle measurement was performed with Fiji (https://fiji.sc/). Statistical analyses and data plotting was performed with R (https://www.r-project.org/). Pairwise Wilcoxon rank sum tests were performed to test the differences of twisting angle between the samples.

## Results

### Loss of Cell-Cell Adhesion Prevents Hypocotyl Twisting in *qua1-1 spr2-2*

To reveal the mechanical conflicts in mutants exhibiting helical growth, we reasoned that disrupting cell-cell adhesion would lead to cell autonomous behavior through the (partial) mechanical uncoupling of cells, and would possibly affect the helical growth of organs. To test that hypothesis, we thus analyzed the *spr2* phenotype in the presence of cell-cell adhesion defects. The *QUA1* gene encodes a glycosyltransferase and mutation in the gene impairs pectin synthesis and cell-cell adhesion (Bouton et al., 2002; Mouille et al., 2007). We generated *qua1-1 spr2-2* lines and observed the hypocotyl phenotype by measuring the twisting angle θ_*T*_. An angle of 0° reveals no twisting, while positive and negative angle values mark left-handed and right-handed twisting, respectively. As reported before, hypocotyls exhibit straight cell files for both WS-4 (Mean θ_*T*_ of −0.72 ± 1.18°, *n* = 12 samples, Figures 2A,F) and Col-0 (Mean θ_*T*_ of 0.09 ± 1.74°, *n* = 12 samples, Figures 2B,G). As expected, in *spr2-2*, hypocotyls exhibited a pronounced right-handed helix of cell files (Mean θ_*T*_ of −9.98 ± 2.85°, *n* = 12 samples, Figures 2D,I). For *qua1-1*, in many cases cell files could not be properly recognized due to the presence of major cell-cell adhesion defects (Figure 2C). However, we could observe cell files in the cortex layer under the epidermis, which revealed no twisting for *qua1-1* (Mean θ_*T*_ of −1.23 ± 1.63°, *n* = 12 samples, Figure 2H). Note that, to allow comparison between genotypes, all quantifications of twisting angles were obtained on that cell layer (Figure 2K, see material and method). Strikingly, we found that in the *qua1-1 spr2-2* double mutant, cell files were straight: *spr2*-induced helical growth was suppressed (θ_*T*_ of −0.23 ± 2.56°, *n* = 12 samples, Figures 2E,J). Pairwise Wilcoxon rank sum test was used to test the differences between these genotypes. While WS-4, Col-0, *qua1-1*, and *qua1-1 spr2-2* were not significantly different from one another, only *spr2-2* was found to be significantly different from all the other genotypes (Figure 2L). This suggests that the mechanical coupling between adjacent cells is indeed required for the production of twisted hypocotyls in *spr2*.

### *qua1-1 spr2-2* Cells Retain the Ability to Undergo Helical Growth

**Figure 3:**
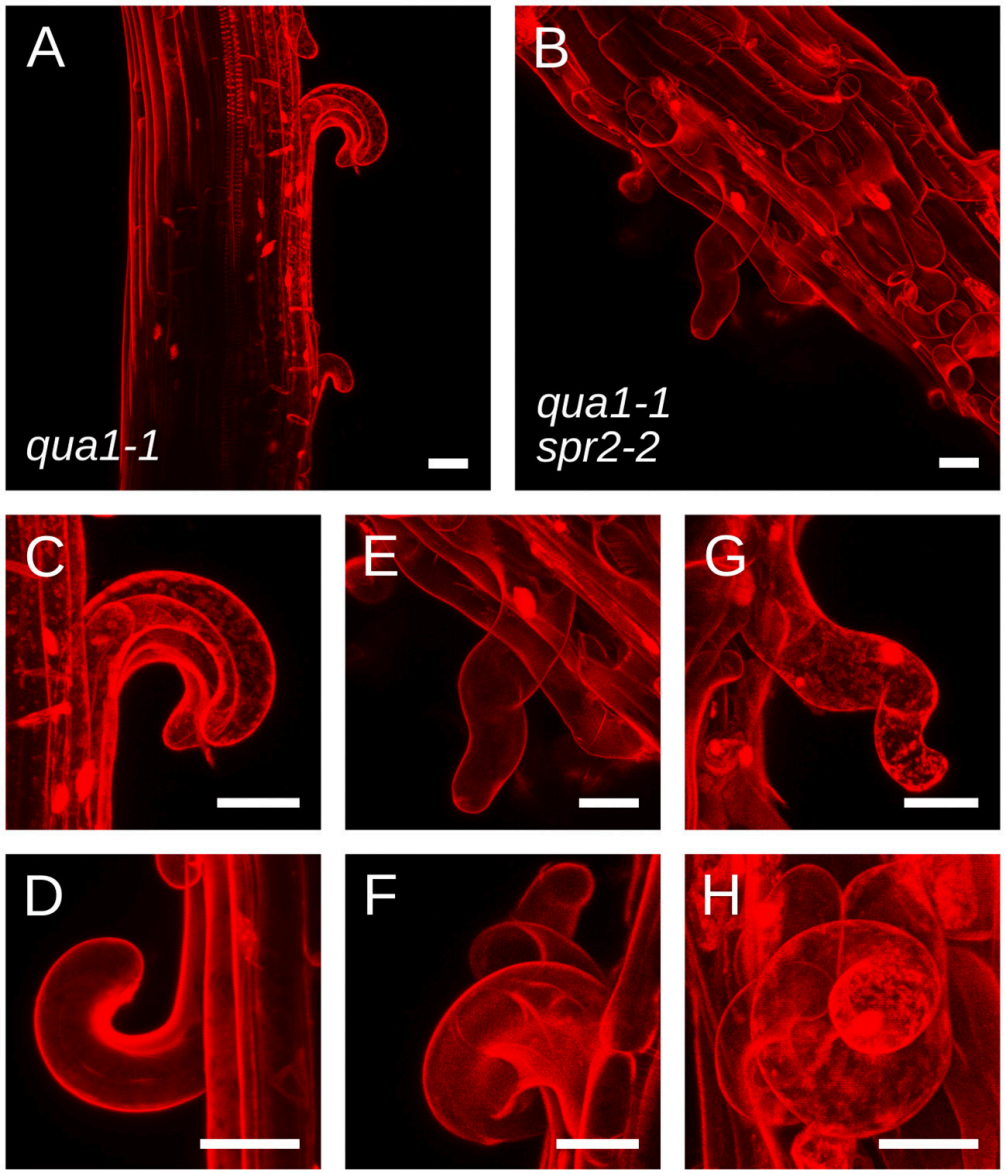
*qua1-1 spr2-2* cells retain the ability to undergo helical growth. **(A–H)** Z-projections (maximal intensity) of confocal stacks from representative, propidium iodide stained, four-day old dark-grown hypocotyls from **(A,C,D)** *qua1-1* and **(B,E–H)** *qua1-1 spr2-2*. Panel C and E are close-ups from **(A,B)** respectively. **(D,F,G,H)** Are additional close-up views from additional *qua1-1* **(D)** and *qua1-1 spr2-2* **(F,G,H)** samples. *qua1-1* cells detach and curl along their longitudinal axis, while in the *qua1-1 spr2-2* background, epidermal cells also detach, but they displayed a clear torsion at the single cell level. Scale bars, 30 μm.

To explain the restoration of straight growth in *qua1-1 spr2-2*, one may invoke alternative hypotheses. For instance, an unknown genetic interaction between *qua1* and *spr2* mutations may compensate the loss of *spr2* activity, e.g., by affecting microtubule dynamics. As mentioned above, the relation between microtubule dynamics and the helical behavior of their arrays is still an open question, so we cannot completely exclude that scenario. Yet, the mechanical uncoupling of adjacent cells in *qua1-1* and *qua1-1 spr2-2* offers the unique opportunity to reveal the contribution of *SPR2* to growth direction in semi-isolated cells. In *qua1-1*, detached epidermal cells curled outward from the hypocotyl, as previously reported (Figures 3A,C,D and Movies S1, S2). More importantly, we observed that these cells did not exhibit twisted growth, they detached and curled along their longitudinal axis, showing that the *qua1* mutation does not affect cell twisting. In the *qua1-1 spr2-2* background, epidermal cells also detached, but they displayed a clear torsion at the single cell level (Figures 3B,E–H and Movies S3,S4). This phenotype could be observed on every *qua1-1 spr2-2* samples. Note that cells had to be sufficiently detached along their axis to exhibit torsion (see Figure 3B in which one cell is largely detached and is twisted, whereas surrounding cells exhibit abnormal morphology but are not twisting on their own as they are not detached from the epidermis). We never observed cells curling “straight” along the longitudinal axis of the hypocotyl in the *qua1-1 spr2-2* line. This strongly suggests that the *spr2* mutation still promotes helical growth in the *qua1-1* background. Therefore, the mechanical uncoupling between adjacent cells in *qua1 spr2-2* allows the relaxation of the local torsional stress by single cell, rather than whole organ, twisting.

### Hypocotyl Twisting in *qua1-1 spr2-2* Is Restored Through the Modulation of Cell-Cell Adhesion Defects

**Figure 4:**
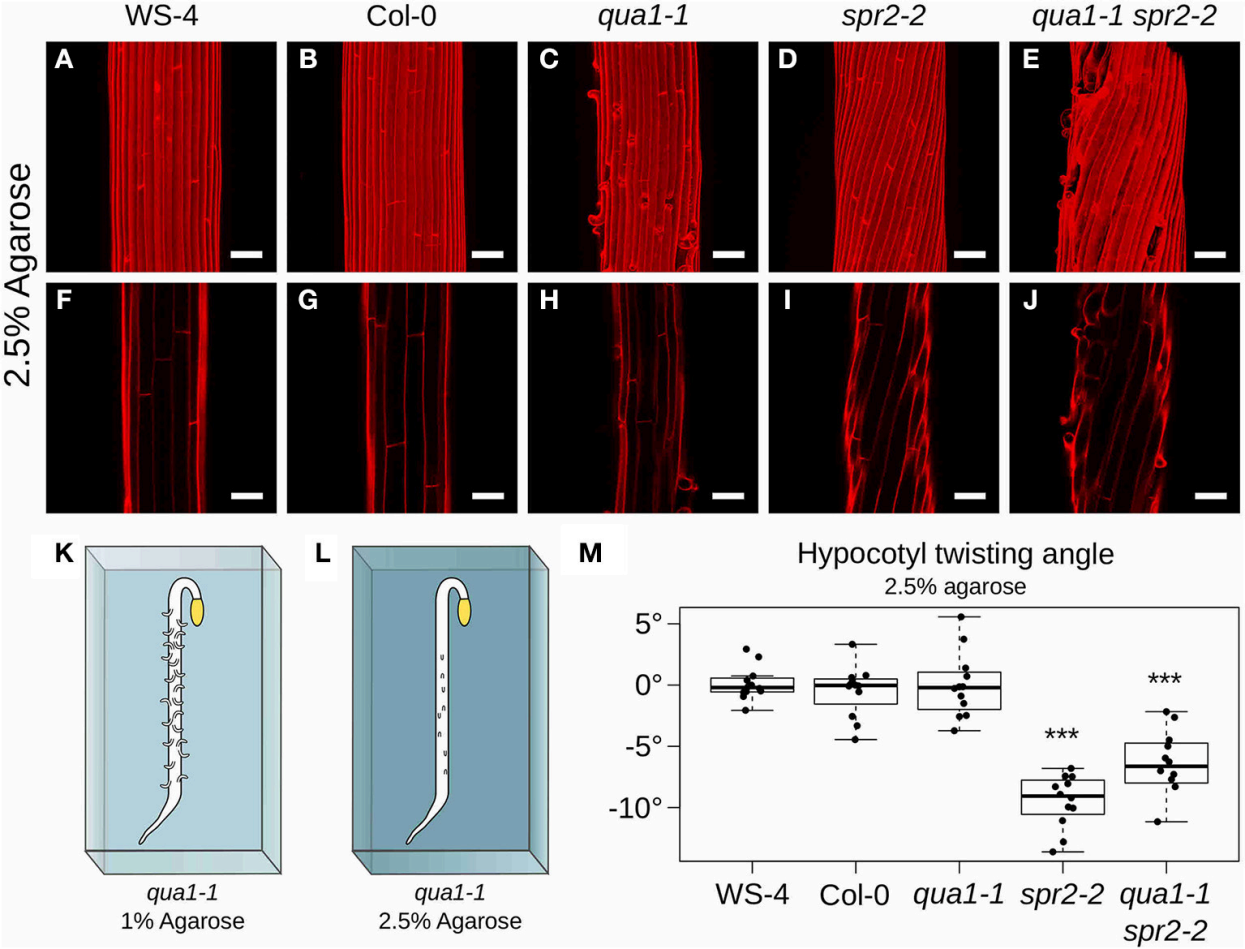
Hypocotyl twisting in *qua1-1 spr2-2* is restored on 2.5% agarose medium. **(A–E)** Z-projections (maximal intensity) of confocal stacks from representative (12 samples observed in 3 biological replicates for each genotype/condition), propidium iodide stained, four-day old dark-grown hypocotyls. **(F–J)** Optical sections from the corresponding stacks. **(A,F)** WS-4, **(B,G)** Col-0, **(C,H)** *qua1-1*, **(D,I)** *spr2-2*, and **(E,J)** *qua1-1 spr2-2*, highlight the restoration of twisting phenotype of *qua1-1 spr2-2* to a degree comparable to that of *spr2-2*. **(K,L)** Rescue of *qua1-1* cell adhesion defect *via* the modulation of the medium water potential. **(K)** Schematic representation of a dark-grown *qua1-1* hypocotyl grown on a 1% agarose medium, and displaying extensive cell adhesion defects (Growth conditions used in Figure 1). **(L)** Schematic representation of a dark-grown *qua1-1* hypocotyl grown on a 2.5% agarose medium, and displaying reduced cell adhesion defects (Growth conditions used in this figure). **(M)** Boxplot of twisting angle values, representing each data point and their distribution for each genotype. An angle of 0° reveals no twisting (straight growth), while positive and negative angle values mark left-handed and right-handed twisting, respectively. Wilcoxon rank sum test ^***^p < 0.0005. Scale bars, 50 μm.

To further confirm that cell-cell adhesion is indeed required for hypocotyl twisting in *spr2-2*, we next undertook to restore adhesion defects in *qua1-1 spr2-2* and check whether hypocotyl twisting would also be restored in these conditions. To do so, we grew the seedlings on medium containing 2.5% agarose, instead of 1% agarose (Figures 4K,L). Indeed, increasing agarose concentration decreases the matrix potential, which in turn affects plant cell mechanics: water availability to the plant and tension in the outer wall are reduced, as previously shown using atomic force microscopy (Verger et al., 2018). In these conditions, cell-cell adhesion defects were largely rescued in *qua1-1*, as previously shown (Verger et al., 2018), consistent with a scenario in which cracks between cells occur only if tension in the epidermis is strong enough to pull cells apart (Figure 4). Therefore, this strategy allowed us to mechanically rescue the adhesion defects in the *qua1-1 spr2-2* double mutant and test its impact on hypocotyl shape.

When seedlings were grown on medium containing 2.5% agarose, hypocotyls still exhibited straight cell files in WS-4 (Mean θ_*T*_ of 0.11 ± 1.36°, *n* = 12 samples, Figures 4A,F), Col-0 (Mean θ_*T*_ of −0.48 ± 2.07°, *n* = 12 samples, Figures 4B,G) and *qua1-1* (Mean θ_*T*_ of −0.02 ± 2.65°, *n* = 12 samples, Figures 4C,H). Similarly the *spr2-2* mutant still exhibited a pronounced right-handed helix of cell files (Mean θ_*T*_ of −9.47 ± 2.14°, *n* = 12 samples, Figures 4D,I). However, twisting growth was almost fully restored in the *qua1-1 spr2-2* background (Mean θ_*T*_ of −6.91 ± 3.53°, *n* = 12 samples, Figures 4E,J). Pairwise Wilcoxon rank sum tests showed that WS-4, Col-0, *qua1-1* were not significantly different from one another, whereas *spr2-2* and *qua1-1 spr2-2* were both significantly different from WS-4, Col-0 and *qua1-1*. Note that *spr2-2* and *qua1-1 spr2-2* were also significantly different from each other. This suggests that the twisting in *qua1-1 spr2-2* is not restored up to the degree observed in *spr2-2* (Figure 4M), also consistent with the observation that cell adhesion defects of *qua1-1* in these conditions are largely rescued but not fully restored (Figure 4E). Nevertheless, it remains that the mechanical “re-coupling” of adjacent cells in *qua1-1* is sufficient to generate a significant impact on twisted growth.

### Cell-Cell Adhesion Defects Suppress Twisted Growth in *qua1-1 spr2-2* Leaves

**Figure 5:**
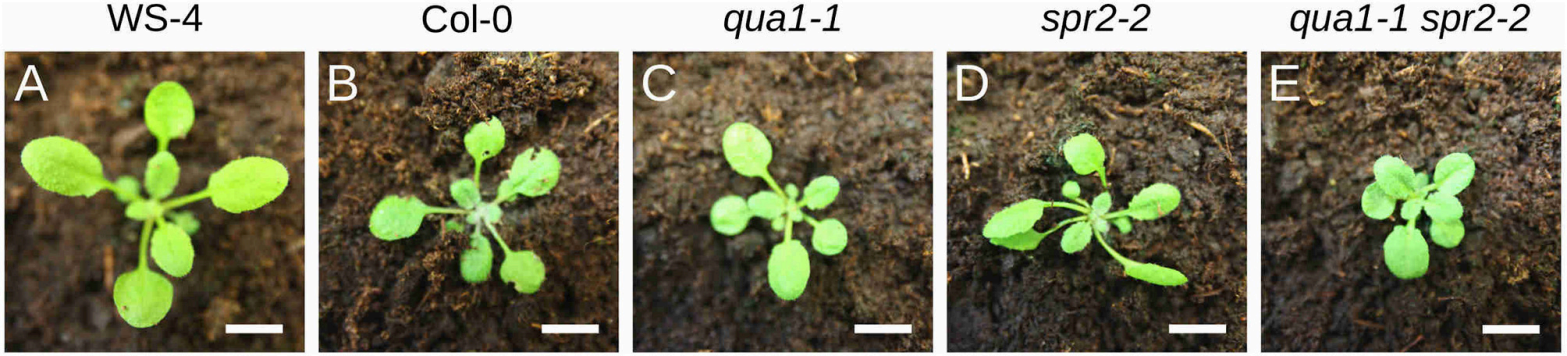
Cell-cell adhesion defects suppress twisted growth in *qua1-1 spr2-2* leaves. **(A–E)** Pictures of 2-week old plants grown on soil. **(A)** WS-4, **(B)** Col-0, **(C)** *qua1-1*, **(D)** *spr2-2*, and **(E)** *qua1-1 spr2-2*, highlight the twisting phenotype of *spr2-2* as compared to the straight growth of the petiole and leaves for the other genotypes. Scale bars, 1 cm.

Because hypocotyl may have a rather specific growth mode (Gendreau et al., 1997) and involving strong tissue tension resulting from mechanical conflicts between the epidermis and inner tissues (Kutschera, 1992; Robinson and Kuhlemeier, 2018), the restoration of straight growth in *qua1-1 spr2-2* may be specific to the hypocotyl. Furthermore, hypocotyl elongation in the *qua1-1 spr2-2* line was reduced, when compared to *spr2-2* mutants (Figures 5D,E) and this may also contribute to the degree of hypocotyl twisting. To test whether the mechanical uncoupling of adjacent cells is sufficient to restore straight growth beyond hypocotyl cells, we grew the double mutant in the greenhouse, on soil. Indeed, *in vitro* plants grow in an atmosphere that is saturated in water, and unless the matrix potential or the osmolarity of the medium is changed, growth conditions are very hypo-osmotic, consistent with the dramatic adhesion defects in *qua1-1* mutants on 1% agar. In fact, in these conditions, viable adult plants cannot be retrieved as the shoot apical meristem also experience massive disorganization and rather resembles a callus-like structure (Verger et al., 2018). Plants that are grown and watered on soil are likely under less hypo-osmotic conditions, simply because the atmospheric hygrometry is not saturated in water. Typically, in our greenhouse, we keep hygrometry at 70%. In such conditions, cell-cell adhesions defects are still present in *qua1-1*, but are not as dramatic as in *in vitro* plants grown on 1% agar. This allowed us to explore the *qua1-1 spr2-2* phenotype beyond the opened cotyledon stage, and in particular in leaves and petioles where tissue twisting is easily detectable. As expected, greenhouse-grown *spr2-2* mutants exhibited twisted leaves (Figure 5). However, such phenotype was not observed in the *qua1-1 spr2-2* double mutant: leaf aspect-ratio was slightly affected, but leaves remained flat (Figure 5). Altogether, these results demonstrate that *spr2-2* mutant cells experience a mechanical conflict that is resolved through organ torsion, via the mechanical coupling of adjacent cells.

## Discussion

Although mechanical conflicts are thought to be widespread in developing organisms, their presence is most often only predicted through computational modeling, or revealed through invasive mechanical alterations such as laser ablations. Here, using the *qua1-1 spr2-2* double mutant with naturally occurring cell-cell adhesion defects and twisted cell growth, we reveal that individual cells tend to undergo torsion, while the restoration of adhesion prevents single cell torsion but leads to organ torsion. Therefore, we provide experimental support for the theory in which organ torsion relaxes the local mechanical conflicts that emerge between adjacent cells with oblique cortical microtubules, and arguably, oblique cellulose microfibrils (Wada and Matsumoto, 2018).

Note that the picture is actually slightly more complex than what is described in Figures 1A,B, notably regarding the actual organization of cortical microtubules and cellulose microfibrils in the hypocotyl. Cortical microtubules and cellulose microfibrils in the epidermis are initially aligned transversely during early and accelerating growth phases of the dark-grown hypocotyl. However they then gradually reorient longitudinally in the outer wall of the epidermis during the rapid and decelerating growth phase, arguably to resist growth and stress from internal tissues (Crowell et al., 2011; Robinson and Kuhlemeier, 2018; Verger et al., 2018), while they remain transverse on the lateral and inner wall faces of the epidermal cells. In fact, such differential mechanical anisotropy on the different faces of the cell could explain the curling phenotype of the detached cells in *qua1-1* (Figures 3A,C,D) as well as the helical shape (rather than simply twisted shape) of the detached cells in *qua1-1 spr2-2* (Figures 3B,E–H). This particular microtubule and cellulose organization remains compatible with the twisting growth model proposed by Wada and Matsumoto (Wada and Matsumoto, 2018). Notably, when a genetic mutation, like *spr2-2*, imposes oblique cortical microtubule orientations, the resistance of longitudinal cellulose microfibrils in the outer cell wall becomes less directional, thus leading to twisting.

Organ twisting is also a good system to analyze the balance between active and passive mechanical response to mechanical conflicts. Indeed, as adjacent cells become separated following cell-cell adhesion defects, the supracellular propagation of mechanical signals also becomes impaired. Typically, tensile stress direction has been proposed to serve as an instructive cue that provides consistent cortical microtubule alignments over several cell files in several plant tissues (Hejnowicz et al., 2000; Hamant et al., 2008; Robinson and Kuhlemeier, 2018). Because the epidermis of aerial organs is under tension in plants, this comes down to a coordinating role of the outer wall that embeds all epidermal cells. Cell-cell adhesion defects generate cracks in that outer wall, disrupting the co-alignment of cortical microtubules (Verger et al., 2018). Therefore, organ torsion in mutants with microtubule defects requires adhesion as a passive mediator of mechanical continuity between adjacent cells, but it may also require adhesion as an active synchronizer of microtubule behavior through mechanical stress propagation. In that respect, the identification of interactions between certain wall receptor kinases and pectin (e.g., Feng et al., 2018), may open the way for an analysis of the interplay between mechanoperception and adhesion in morphogenesis.

Cell-cell adhesion defects also destroy plasmodesmata connections, and thus alter the possibility to have large symplastic domains with consistent growth properties. In that scenario, isolated cells in adhesion mutants may grow independently from their neighbors, as clearly shown by the detached *qua1-1 spr2-2* mutant cell morphology. This may have two effects: cell growth heterogeneity may increase because neighboring cells would not mutually constrain their growth anymore, and this would likely result in distorted organ shapes. In an alternative scenario, growth heterogeneity may decrease, either because the presence of adjacent cells rather fuels growth heterogeneity, as observed in shoot apical meristems (Uyttewaal et al., 2012), or because the supracellular averaging of individual cells growing at different speed may produce more reproducible organs than large sectors of cells growing at different speed, as shown in sepals (Hong et al., 2016). The ambivalent nature of mechanical conflicts in growth heterogeneity has recently been analyzed in computer simulations (Fruleux and Boudaoud, 2019). In a more complex scenario, plasmodemata may have a direct role in organ twisting. Although this is less likely and still largely hypothetical, carpels were shown to twist in the *quirky* mutant (Trehin et al., 2013) and the QUIRKY protein localizes to plasmodesmata (Vaddepalli et al., 2014). When confronted to our results, these alternative scenarios are not exclusive. Yet, the idea that cell-cell adhesion primarily disrupts the passive relaxation of local mechanical conflicts is by far the most parsimonious in the case of organ torsion.

Finally, we focused here on the *spiral2* mutant with a fixed handedness, which is usually the case for mutants affected in microtubule functions. There are other ways to induce organ twisting in Arabidopsis. In particular, mutants affected in auxin response or transport can exhibit twisted organs too, although the handedness is not fixed in such cases (Ishida et al., 2007b). More generally, organ twisting is widespread in Angiosperms, and this offers several adaptative and evolutive advantages (Smyth, 2016). For instance, growing organs can rapidly twist in order to reorient relative to light source or gravity field in a process called “helical tropism” (Borchers et al., 2018). Thin vertical leaves of Typha sp. tend to twist and this has been associated to increased stability and reduced bending of the leaf in response to its own weight (Schulgasser and Witztum, 2004); twisted awns of wheat seeds contribute to their dispersal (Elbaum et al., 2007); tendrils twist through contraction of internal tissues, thereby allowing mechanical support (Gerbode et al., 2012). Understanding organ twisting may thus also have important ecological and developmental implications.

## Author Contributions

SV and ML performed the experiments. SV analyzed the results. SV and OH wrote the article. OH secured funding for this project.

### Conflict of Interest Statement

The authors declare that the research was conducted in the absence of any commercial or financial relationships that could be construed as a potential conflict of interest.

## Supplementary Material

Movie S1 Cell curling in *qua1-1*. 360 degree rotation from the sample presented in Figure 3C.

Movie S2 Cell curling in *qua1-1*. 360 degree rotation from the sample presented in Figure 3D.

Movie S3 Cell curling in *qua1-1 spr2-2*. 360 degree rotation from the sample presented in Figure 3E.

Movie S4 Cell curling in *qua1-1 spr2-2*. 360 degree rotation from the sample presented in Figure 3G.
